# Western blot data using two distinct anti-*O*-GlcNAc monoclonal antibodies showing unique glycosylation status on cellular proteins under 2-deoxy-d-glucose treatment

**DOI:** 10.1016/j.dib.2016.12.001

**Published:** 2016-12-09

**Authors:** Tetsuya Okuda

**Affiliations:** Bio-design research group, Bioproduction research Institute, National Institute of Advanced Industrial Science and Technology (AIST), Central 6, 1-1-1 Higashi, Tsukuba 305-8566, Ibaraki, Japan

**Keywords:** 2-deoxy-d-glucose, *O*-GlcNAcylation, Sp1, Teratocarcinoma

## Abstract

Protein modification by *O*-linked *N*-acetylglucosamine (*O*-GlcNAcylation) is one of the post transcriptional modifications occurring on cellular proteins. This paper provides a data set relating to the *O*-GlcNAcylation of cellular proteins detected by RL2 and CTD110.6 antibodies, which are commonly used for detection of protein *O*-GlcNAcylation, in 2-deoxy-d-glucose (2DG)-treated human teratocarcinoma NCCIT cells in support of the research article entitled “A novel, promoter-based, target-specific assay identifies 2-deoxy-d-glucose as an inhibitor of globotriaosylceramide biosynthesis” (Okuda et al., 2009) [1]. The main article described a suppressive effect of 2DG on an Sp1 target gene in NCCIT cells and discussed the relationship between the effect of 2DG and *O*-GlcNAcylation status of Sp1. The data in this paper complements this relationship by Western blotting and clearly showed that the 2DG treatment increased *O*-GlcNAcylation of cellular proteins in NCCIT cells, whereas the RL2 and CTD110.6 epitopes were detected in a different manner. The RL2 epitope was detected on Sp1 during 2DG treatment, and the level was transiently increased at 24 h. In contrast, the CTD110.6 epitope became detectable on Sp1 over 72 h after 2DG treatment, and then the other proteins containing CTD110.6 epitopes also appeared in the cell lysates and the anti-Sp1 antibody precipitates.

**Specifications Table**

**Table:** 

Subject area	*Biology*
More specific subject area	*2-deoxy-d-glucose treatment, O-GlcNAcylation of cellular proteins*
Type of data	*Western blotting*
How data was acquired	*Western blotting using a chemiluminescent substrate (ECL Prime Western Blotting Detection Reagent, GE Healthcare UK Ltd.) and an image analyzer (Light-Capture ATTO Co.).*
Data format	*Raw data for Western blotting*
Experimental factors	*Cells were treated with 2-deoxy-d-glucose (2DG) and cellular proteins were analyzed by ECL Prime Western Blotting system.*
Experimental features	*Human teratocarcinoma NCCIT cells were incubated with a culture medium supplemented with 10 mM 2DG for 24–168 h. Protein extracts of the cells and the immunoprecipitates of anti-Sp1 antibody (D4C3) were subjected to Western blotting.*
Data source location	*Bioproduction research Institute, National Institute of Advanced Industrial Science and Technology (AIST), Central 6, Tsukuba*
Data accessibility	*The data are provided in this article.*

**Value of the data**

• The data clearly showed a unique effect of 2DG on the *O*-GlcNAcylation of cellular proteins, specifically the 2DG effect on *O*-GlcNAcylation of Sp1 can be of value for researchers from related fields.
• These data can be compared to other scientific data addressing 2DG effects on various cells and tissues.
• Protocols providing here support other researchers to execute the optimum assay for the evaluation of Sp1 and *O*-GlcNAcylation.

## 1 Data

**Fig. 1:**
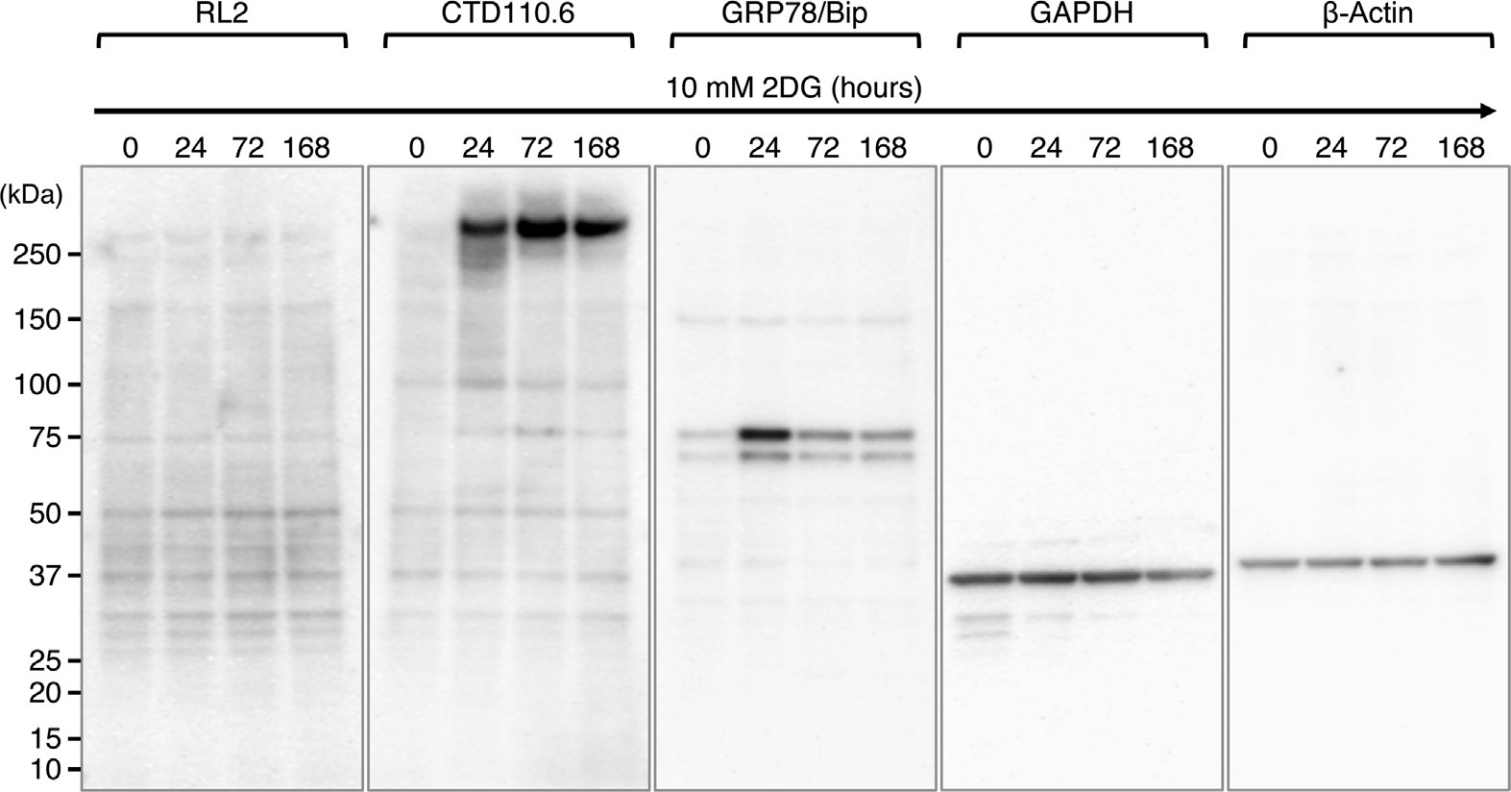
Western blot analysis of *O*-GlcNAcylated proteins in 2DG-treated NCCIT cells. The whole cell lysates of NCCIT cells treated with 2DG for the indicated times (0, 24, 72, or 168 h) were immunoblotted with anti-*O*-GlcNAc antibodies (RL2 or CTD110.6). Since the 2DG behaves as a glucose starvation mimetic in cells [1], the levels of a marker for glucose starvation (GRP78/Bip) was indicated as an internal control. The GRP78/Bip is known to be transiently increased under glucose starvation [5]. In NCCIT cells, the transient increase of GRP78/Bip was confirmed at 24 h after 2DG treatment. GAPDH and β-Actin levels were also indicated for internal controls.

**Fig. 2:**
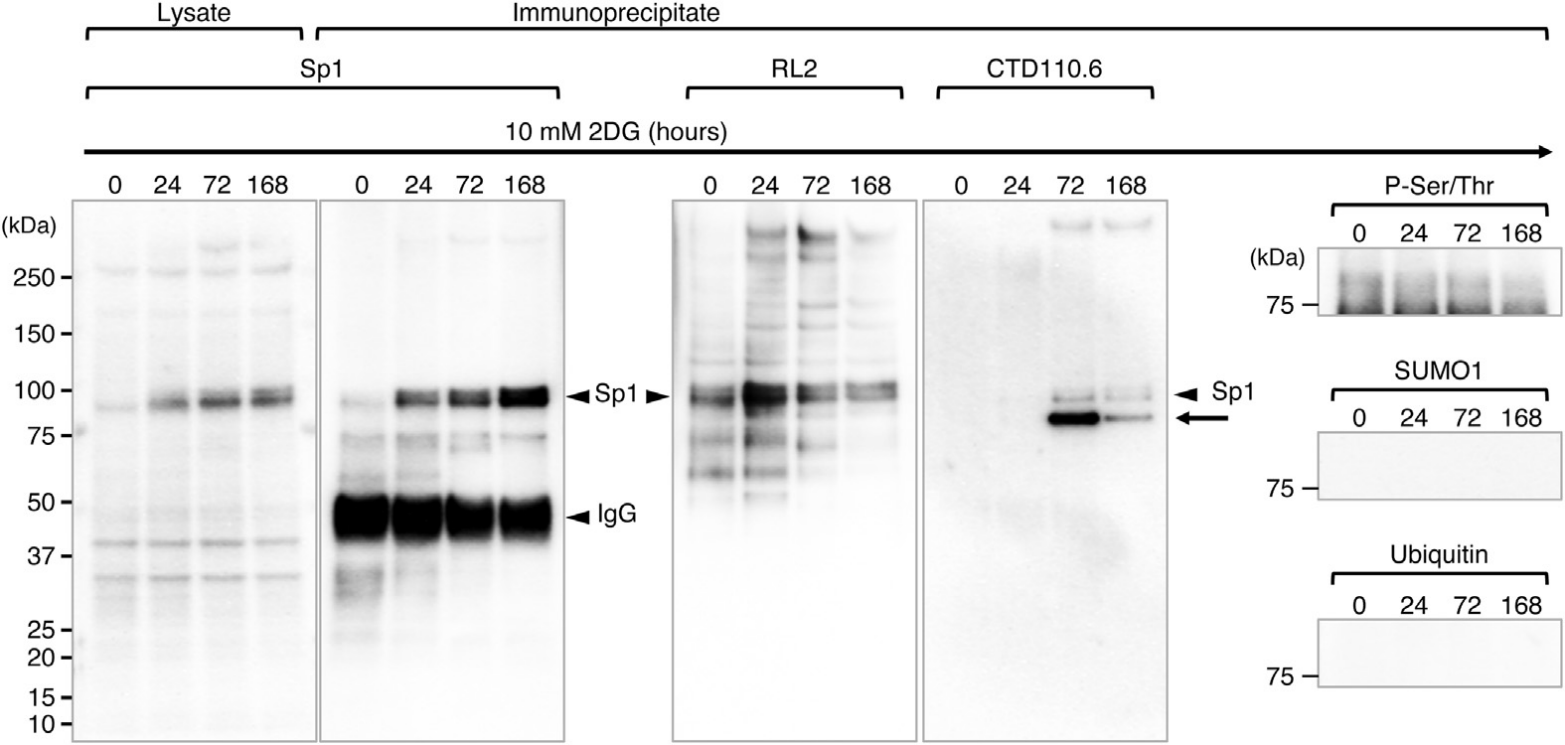
Western blot analysis of Sp1 protein in 2DG-treated NCCIT cells. The whole cell lysates of NCCIT cells treated with 2DG for the indicated times (0, 24, 72, or 168 h) were precipitated with an anti-Sp1 antibody (D4C3), and the precipitated proteins were immunoblotted with D4C3, anti-*O*-GlcNAc antibodies (RL2 or CTD110.6), anti-Phosphoserine/threonine (P-Ser/Thr), anti-SUMO1, or anti-Ubiquitin. These transcriptional modifications were observed in Sp1 proteins [6]. Among these modifications, only *O*-GlcNAcylation was clearly detected in the D4C3 precipitates. The Sp1 levels in whole cell lysates were shown as a reference (left panel).

Status of *O*-GlcNAcylation of proteins in 24–168 h 2DG-treated NCCIT cells were analyzed by Western blotting using two distinct anti-*O*-GlcNAc monoclonal antibodies, RL2 [2] and CTD110.6 [3]. The data indicate that 2DG treatment increased the *O*-GlcNAcylation of cellular proteins in NCCIT cells, whereas RL2 and CTD110.6 epitopes were detected in a different manner in whole cell lysates of NCCIT cells (Fig. 1) and anti-Sp1 antibody precipitates (Fig. 2).

Although RL2 epitopes were hardly detected in cellular proteins of whole cell lysates, these were detected on Sp1 during 2DG treatment and the level was transiently increased at 24 h (Fig. 2). Some of minor protein bands containing RL2 epitopes were also detected in the anti-Sp1 antibody precipitates. CTD110.6 blot indicates an over 250 kDa protein containing CTD110.6 epitope is strongly induced by 2DG treatment (Fig. 1). CTD110.6 epitopes also become detectable on Sp1 over 72 h 2DG treatment (Fig. 2). At the same time, a protein containing CTD110.6 epitope is coimmunoprecipitated with Sp1 (Fig. 2, arrow). In addition, we found that 2DG treatment continuously increases Sp1 levels in NCCIT cells.

Sp1 bears multiple *O*-GlcNAc residues [4] and RL2 and CTD110.6 epitopes are increased on Sp1 with different kinetics in NCCIT cells under 2DG treatment, indicating that RL2 and CTD110.6 recognize different *O*-GlcNAc residues on Sp1. The amounts of *O*-GlcNAc residues on Sp1 are relatively low at 168 h, indicating that long time 2DG treatment ultimately decreases *O*-GlcNAcylation in NCCIT cells.

## 2 Experimental design, materials and methods

### 2.1 Cell culture

Human teratocarcinoma NCCIT cells were obtained from the American Type Culture Collection (Manassas, VA, USA), and were maintained in RPMI-1640 medium supplemented with 10% fetal bovine serum and 2 mM l-glutamine. NCCIT cells were seeded at 2×10^6^ cells on culture dishes (100 mm in diameter) and incubated for 24 h prior to stimulation with 2DG. After incubation, the medium was replaced with fresh culture medium containing 10 mM 2DG and incubated for an additional 24–168 h. The medium was replaced every 48 h. Cells were cultured at 37 °C in a humidified atmosphere containing 5% CO_2_.

### 2.2 Preparation of whole cell lysate

NCCIT cells in 100 mm dishes were lysed using 0.5 ml of RIPA buffer (PBS with 1% Nonidet P-40, 0.5% sodium deoxycholate, 0.1% SDS) supplemented with 50 μM PUGNAc and 0.1 mM phenylmethylsulfonyl fluoride, and a proteinase inhibitor cocktail (Complete mini EDTA-free; Roche, Basel, Switzerland).

### 2.3 Immunoprecipitation

The whole cell lysates (0.2 ml) were incubated with 4 μl (1 μg) of rabbit anti-Sp1 monoclonal antibody (D4C3, Cell Signaling Technology, Danvers, MA) at 4 °C overnight, and subsequently incubated with 20 μl of 50% protein A sepharose bead slurry (GE Healthcare) at 4 °C for 3 h. The samples were centrifuged at 14,000 g for 1 min at 4 °C, and the precipitates (beads) were washed five times with 0.5 ml of wash buffer (20 mM Tris–HCl pH 7.5, 150 mM NaCl, 1 mM EDTA, 1 mM EGTA, 1% Triton X-100, 2.5 mM sodium pyrophosphate, 1 mM β-glycerophosphate, 1 mM Na_3_VO_4_, 1 μg/ml leupeptin, 1 mM phenylmethylsulfonyl fluoride, and 50 μM PUGNAc) and were finally resuspended in 20 μl of SDS-PAGE sample buffer.

### 2.4 Western blotting

The whole cell lysates (5 μg of total protein) and anti-Sp1 antibody precipitates were separated by SDS-PAGE, and the proteins in the gel were transferred onto Immobilon-P PVDF membranes (Millipore, Billerica, MA) by electroblotting at a constant current of 90 mA for 1 h. After blotting, the membrane was incubated with primary antibody and subsequently incubated with a horseradish peroxidase-linked secondary antibody. Antibody binding was detected using ECL Prime Western Blotting Detection Reagent (GE Healthcare UK Ltd., Buckinghamshire, UK) and an image analyzer (ATTO Light-Capture, ATTO, Tokyo, Japan). The reproducibility of result was confirmed by three independent experiments (*n*=3). The primary antibodies used were mouse anti-*O*-GlcNAc monoclonal antibodies (RL2, Thermo Fisher Scientific, Waltham, MA; CTD110.6, Cell Signaling Technology), rabbit anti-Sp1 monoclonal antibody (D4C3, Cell Signaling Technology), rabbit anti-GRP78/Bip monoclonal antibody (C50B12, Cell Signaling Technology), rabbit anti-GAPDH monoclonal antibody (D16H11, Cell Signaling Technology), rabbit anti-β-Actin polyclonal antibody (#4967, Cell Signaling Technology), rabbit anti-Phosphoserine/threonine polyclonal antibody (ab17464, abcam, Cambridge, UK), mouse anti-SUMO1 monoclonal antibody (21C7, BostonBiochem, Cambridge, MA), and mouse anti-Ubiquitin monoclonal antibody (P4D1, Cell Signaling Technology).
